# Childhood trauma and treatment outcomes in short-term psychodynamic and cognitive behavioral therapy for adult depression

**DOI:** 10.3389/fpsyt.2025.1692277

**Published:** 2025-11-07

**Authors:** Anders Malkomsen, Randi Ulberg, Toril Dammen, Julie Horgen Evensen, Benjamin Hummelen, André Løvgren, Kåre Osnes, Theresa Wilberg, Jan Ivar Røssberg

**Affiliations:** 1 Division of Mental Health and Addiction, Oslo University Hospital, Oslo, Norway; 2 Institute of Clinical Medicine, University of Oslo, Oslo, Norway; 3 Nydalen Outpatient Clinic, Oslo University Hospital, Oslo, Norway; 4 Division of Mental Health and Substance Abuse, Diakonhjemmet Hospital, Oslo, Norway

**Keywords:** childhood trauma, depression, cognitive behavioral therapy, psychodynamic therapy, treatment outcome, moderation

## Abstract

Childhood trauma (CT) is a known risk factor for major depressive disorder (MDD), yet its implications for treatment selection remain unclear. This exploratory randomized controlled trial (N = 100) compared short-term psychodynamic psychotherapy (STPP) and cognitive behavioral therapy (CBT) in adults with MDD treated in outpatient clinics. CT was assessed with the Childhood Trauma Questionnaire–Short Form (CTQ-SF), and depressive symptoms were measured using the Beck Depression Inventory-II (BDI-II) and the Hamilton Depression Rating Scale (HDRS) at baseline and after 28 weeks of therapy. Linear mixed-model analyses showed that CT was associated with higher depression severity at the start of treatment. No significant predictive effects of CT were found for treatment outcome or for moderation of the comparative effectiveness of CBT and STPP. Emotional abuse was initially associated with greater improvement in STPP compared to CBT on the HDRS, but the effect did not remain significant after correction for multiple testing. These findings suggest that both CBT and STPP are suitable options for patients with MDD and a history of CT.

## 1 Introduction

Depression is among the most prevalent mental disorders, a leading cause of disability, and a major contributor to the global disease burden (1). Childhood trauma (CT), defined as emotional and physical neglect or emotional, physical, or sexual abuse before the age of eighteen, is a significant risk factor for both developing major depressive disorder (MDD) and experiencing a more severe and chronic course (2–8). Childhood emotional abuse and neglect more than double the risk for a depressive disorder (9), with a graded relationship suggesting that the risk increases with more adverse events (10, 11). The impact of childhood maltreatment varies by type, with emotional abuse and neglect showing a stronger association with depressive symptoms than sexual or physical abuse (12–14). Studies suggest that depression in patients with CT may represent a distinct subtype of depression (15), neurobiologically characterized by blunted cortisol response to stress, diminished hippocampal gray area and exaggerated amygdala response to negative information (16). This may contribute to more severe neurovegetative and psychomotor symptoms (17) and to more severe depressive symptoms at baseline in patients with CT (3).

Previous meta-analyses have suggested that CT is associated with poorer treatment outcomes for depression, including a higher risk of non-response (2, 4). A meta-analysis from 2022 by Kuzminskaite et al. (18), including 29 studies – 28 with treatment durations between 5 and 30 sessions – found no significant difference in treatment response between patients with and without trauma. This finding was contested by Danese & Uher (19), who pointed to potential biases, study heterogeneity, and the absence of long-term relapse analysis, suggesting that tailored interventions may still be warranted for trauma survivors. In their defense, Kuzminskaite et al. (20) reaffirmed their results, arguing that patients with CT do indeed benefit similarly from standard treatments. Nevertheless, they acknowledged the limitation of selection bias, as nearly half of the relevant studies were not available for inclusion in their analysis.

While psychotherapy and medication are generally considered equally effective for depression (21), findings in patients with CT have been mixed. Gruhn et al. (22) found greater symptom improvement with antidepressants compared to cognitive psychotherapy, whereas Nemeroff et al. (23) reported superior outcomes with psychotherapy over antidepressant monotherapy, with combination therapy offering only marginal additional benefits. Similarly, Williams et al. (24) found CT to predict poorer antidepressant response, while Zobel et al. (25) showed that patients with CT benefitted significantly more from a combination of interpersonal dynamic psychotherapy and antidepressants than from medication alone. Collectively, these findings point to psychotherapy as an important treatment option for depressed patients with CT, with potential for enhanced effectiveness when combined with medication.

The optimal choice of psychotherapeutic approach for depressed patients with CT remains uncertain. Both cognitive-behavioral therapy (CBT) and short-term psychodynamic psychotherapy (STPP) are effective psychotherapeutic treatments for MDD (26–32), despite their significant theoretical and practical differences. STPP emphasizes how past experiences (especially childhood trauma) influence current relationships and unconscious processes (33). Therapists address relational patterns, defense mechanisms, and transference dynamics to foster insight and change. Because early trauma often leads to enduring interpersonal difficulties (34), psychodynamic therapies have been hypothesized to be especially effective in CT, as they target precisely these past experiences and relational processes (35–41). In contrast, CBT treats depression by modifying maladaptive cognitions and behaviors, based on the premise that emotions and actions are shaped by cognitive structures (42). Therapists help patients identify and challenge negative thoughts, adopt alternative perspectives, and engage in anti-depressive activities. Cognitive research has identified that childhood trauma contributes to the development of maladaptive schemas (43), and to automatic negative thoughts about oneself (44), which may be more directly addressed in CBT (45).

Existing comparative research offers some insights into the effectiveness of CBT and STPP in depressed patients with childhood trauma. In a study from 2024 on chronically depressed patients with CT, Krakau et al. (39) found that open-ended and long-term psychoanalytic therapy (PAT) was more effective than CBT after five years, with 242 sessions in PAT and 59 in CBT. A moderating effect was observed for the CTQ total score, as well as the subscales of sexual abuse and family inconsistency. Importantly, this difference was limited to patients with higher CTQ scores, as both treatments yielded comparable outcomes at lower levels of trauma. In 2012, Harkness et al. (40) compared shorter-term CBT and interpersonal psychotherapy (IPT), with 16 weeks of treatment, and found a moderating effect as depressed patients with CT benefited more from CBT. Again, this difference was only observed in patients with “severe maltreatment”, which may indicate that the severity of trauma plays an important role as a moderator. The conflicting results of these two studies also indicate that the duration of treatment may influence the moderation effect. A study by Heinonen et al. (41) from 2018 gives further evidence for this effect as it examines the impact of CT on depressed and anxious patients by comparing solution-focused therapy (SFP), which shares some similarities with CBT, with long-term psychodynamic therapy (LTPP) and short-term psychodynamic psychotherapy (STPP). They found that in STPP (15 sessions), greater childhood unhappiness was associated with fewer depressive symptoms shortly after therapy, but in LTPP (252 sessions), contrary to their predictions, higher scores correlated with worse outcomes at the 12-month follow-up. In SFP (10 sessions), higher scores did not predict poorer outcomes, contrary to their hypothesis. This highlights the complexity of tailoring treatments for patients with CT, emphasizing the need for more studies.

While quantitative research provides essential insights into symptom reduction and treatment effectiveness, qualitative research is crucial for capturing the lived experiences of depressed patients with CT. Nilsson et al. (46) found that patients satisfied with PDT often attributed their improvement to getting to the root of their problems or working through trauma. Similarly, Valkonen et al. (47) reported that patients who framed their distress in terms of past trauma (life-historical narrative) were more likely to experience symptom improvement and narrative development when treated with long-term PDT rather than CBT. This may reflect the emphasis in PDT on exploring past experiences, which could be particularly relevant for patients with CT. Together, these qualitative findings add support to the hypothesis that PDT/STPP may offer unique therapeutic benefits for patients with childhood trauma.

In the current study, patients with MDD were provided either CBT or STPP. The two approaches were found to be equally effective in improving depressive symptoms, anxiety, quality of life and psychosocial functioning (32). Childhood trauma was measured by Childhood Trauma Questionnaire – Short Form (CTQ-SF). Although previous research has examined the association between childhood trauma and treatment outcomes in depression, much remains unclear, including whether, and for whom, specific therapeutic approaches may offer differential benefits. Aiming to fill this gap, the present study examines three research questions:

1. Are higher CTQ-SF scores associated with greater depressive symptom severity at baseline? We hypothesize that higher CTQ-SF scores will be positively associated with greater depressive symptom severity at the start of treatment.
2. Do higher CTQ-SF scores predict less depressive symptom improvement during treatment? We hypothesize that higher CTQ-SF scores will be associated with poorer treatment outcomes.
3. Do CTQ-SF scores moderate the comparative effectiveness of STPP and CBT for depressive symptoms? We hypothesize that STPP will be more effective than CBT for patients with higher CTQ-SF scores, while outcomes will be comparable at lower scores.

## 2 Methods

### 2.1 Design, setting and recruitment

This study is part of the Mechanisms of Change in Psychotherapy (MOP) project and builds on the pre-registered MOP protocol (48), where CT was listed as one of several moderators that would be explored. The patients were randomly assigned to receive 19 sessions of CBT over a 28-week period or 28 weekly sessions of STPP. The randomization was not stratified, and no block randomization was applied. While 100 patients were included, one patient did not complete the CTQ-SF form and was excluded from the analysis, resulting in a final sample in the current study of 99 patients. After 28 weeks, 11 CBT patients and 8 STPP patients were lost to follow-up. For more details on the study design, we refer to a previous publication (32).

The treatment took place at two public psychiatric outpatient clinics in Oslo, at Nydalen, Oslo University Hospital, and Vinderen, Diakonhjemmet Hospital. Both clinics treat patients with a wide range of mental illnesses. The patients were recruited consecutively as they were referred to the outpatient clinics with depressive symptoms as their main reason for referral. Patients were assessed for symptom disorders according to DSM-IV criteria using the Mini International Neuropsychiatric Interview 6.0.0 (MINI) (49). Personality disorders were assessed using the Structured Clinical Interview for DSM-IV Axis II Personality Disorders (SCID-II) (50). In addition to MDD, the inclusion criteria were age 18–65 years, an ability to understand, write and speak a Scandinavian language, and willingness to give informed consent. Exclusion criteria were current or past neurological illness, psychotic disorders, traumatic brain injury, bipolar disorder type 1, current alcohol and/or substance dependence disorders, developmental disorders, and intellectual disability. Patients with bipolar I disorder were excluded because the study focused on psychotherapy for unipolar major depression.

The study was reviewed and approved by the Central Norway Regional Committee for Medical and Health Research Ethics (REC South East, reference 2016/340). The trial was registered at ClinicalTrials.gov (Identifier: NCT03022071). All participants provided written informed consent prior to participation, in accordance with the Declaration of Helsinki.

### 2.2 Patient sample description

Clinical characteristics and demographic information for the CBT and STPP groups are shown in **Table 1**. There were no significant differences between the groups on any of the clinical measures. Emotional neglect was the most common form of adversity, reported to some degree by 60% of patients (34% low, 10% moderate, 16% severe). Emotional abuse followed, reported by 38% of patients (25% low, 9% moderate, 4% severe). Physical neglect was less common, reported by 21% of patients (10% low, 7% moderate, 4% severe), while physical abuse was reported by 4% (2% low, 1% moderate, 1% severe). Sexual abuse was reported by 9% (5% low, 3% moderate, 1% severe). Only seven patients reported that they had not experienced any form of CT. The patient population of the current study has levels of childhood trauma comparable to those previously reported in psychiatric populations (51) and patients with depression (39), although there were slightly less patients with sexual and physical abuse.

**Table 1:** Demographic and clinical characteristics of the patient sample, stratified by short-term psychodynamic psychotherapy (STPP) and cognitive behavioral therapy (CBT).

Baseline characteristics	STPP (N = 50)	CBT (N = 49)
	*% (N)*	*% (N)*
Age, mean (s.d.)	30.4 (8.0)	31.9 (9.3)
Gender		
Female	60 (30)	59 (29)
Male	40 (20)	41 (20)
Antidepressant use	36 (18)	49 (24)
Psychotherapy experience ^a^	58 (29)	67 (33)
Previous admissions ^b^	8 (4)	6 (3)
Ethnicity		
European	100 (50)	94 (46)
Other	0 (0)	6 (3)
Marital status		
Married/partner	40 (20)	39 (19)
Unmarried/no partner	60 (30)	61 (30)
Education level ^c^		
Very low (< 3 years)	6 (3)	10 (5)
Low (≥ 3 years)	28 (14)	29 (14)
Intermediate (≥ 6 years)	38 (19)	53 (26)
High (≥ 9 years)	28 (14)	8 (4)
Job status		
Working	62 (31)	74 (36)
Student	24 (12)	20 (10)
Social security benefits	14 (7)	6 (3)
Axis 1 disorders		
Major depressive disorder	100 (50)	100 (49)
Recurrent depressive disorder	64 (32)	69 (34)
Bipolar II disorder	2 (1)	0 (0)
Dysthymia	2 (1)	0 (0)
Panic disorder	12 (6)	14 (7)
Panic disorder w/agoraphobia	14 (7)	14 (7)
Agoraphobia w/o panic disorder	10 (5)	8 (4)
Post-traumatic stress-disorder (PTSD)	4 (2)	2 (1)
Generalized anxiety disorder	4 (2)	4 (2)
Social phobia	14 (7)	16 (8)
Obsessive-compulsive disorder	0 (0)	2 (1)
Personality disorders		
Avoidant	18 (9)	12 (6)
Dependent	0 (0)	2 (1)
Obsessive-compulsive	2 (1)	0 (0)
Paranoid	4 (2)	4 (2)
Personality disorder NOS	6 (3)	6 (3)
HDRS baseline, mean (s.d.)^d^	17.9 (5.5)	18.1 (5.6)
BDI-II baseline, mean (s.d.)^e^	26.5 (7.6)	28.5 (7.6)
CTQ-SF total, mean (s.d.)^f^	36.0 (9.46)	39.0 (9.46)
CTQ-SF Emotional abuse, mean (s.d)	8.0 (2.9)	8.9 (3.5)
CTQ-SF Emotional neglect, mean (s.d)	11.1 (5.2)	12.3 (5.0)
CTQ-SF Physical abuse, mean (s.d.)	5.2 (0.7)	5.6 (1.5)
CTQ-SF Physical neglect, mean (s.d.)	6.5 (2.9)	6.5 (2.3)
CTQ-SF Sexual abuse, mean (s.d.)	5.2 (1.0)	5.7 (1.5)
CTQ-SF Minimization-denial, mean (s.d.)	8.3 (3.3)	8.3 (3.4)

^a^Previous psychotherapy experience (≥ 1 x week). ^b^Previous admission in psychiatric hospital. ^c^Education after primary school (ten years). ^d^Independent samples t-test (ISTT), p-value = 0.84. ^e^ISTT = 0.19. ^f^ISTT = 0.14. NOS, Not otherwise specified; CTQ-SF, Childhood Trauma Questionnaire – Short Form; HDRS, Hamilton Depression Rating Scale; BDI-II, Beck Depression Inventory II.

### 2.3 Therapists, treatment, supervision and fidelity

A total of 18 therapists participated in the treatment, including 12 women and six men. The group consisted of nine psychologists, six psychiatrists, and three psychiatric nurses. Therapists were highly experienced in both groups, with an average of about 14 years of practice and at least two years of formal training in their approach.

The CBT treatment consisted of 16 weekly sessions followed by three monthly booster sessions, and was based on Aaron Beck’s book “Cognitive Therapy of Depression” (42). Therapists were told to begin each session with a mood assessment, a review of homework, and to collaboratively set an agenda. Sessions were to conclude with a summary, as well as personalized homework assignments to address ongoing issues. Interventions included Socratic questioning; using schematic models to explore thoughts, emotions, and behaviors; challenging automatic thoughts; behavioral activation; and addressing thinking traps. Initial sessions (1–3) focused on building a therapeutic alliance, setting goals, and developing case formulations linking past experiences to current dysfunctions. Subsequent sessions (4–16) aimed at modifying dysfunctional cognitions and behaviors. Booster sessions (16–19) were designed to consolidate progress and enhance relapse prevention.

The STPP treatment consisted of 28 weekly sessions based on Glen O. Gabbard’s book “Long-term Psychodynamic Psychotherapy” (33), applied to a 28-session time frame as described by Cregeen et al. (52). Therapists were instructed to reduce depressive symptoms by addressing unconscious processes, childhood influences, transference, and defense mechanisms, adapted in a flexible manner. Case formulations were completed within the first three sessions, focusing on symptoms, life events, and maintaining factors. The major themes were revisited between sessions 8–20, with termination preparation starting in session 20. The final sessions emphasized current struggles and post-treatment application of the insights gained in therapy. We deliberately offered more sessions in STPP to reflect routine clinical practice, where psychodynamic therapies are typically offered over more sessions than CBT. Importantly, our statistical analyses indicated that the number of sessions was not associated with treatment outcome (32).

All therapy sessions were video-recorded, and treatment fidelity was monitored by experienced supervisors through weekly one-hour group supervision for STPP and bi-weekly two-hour group supervision for CBT. Supervision focused on the initial treatment phase, case formulation, individual treatment strategies, and therapy termination. The treatments were reliably discriminated by using the Comparative Psychotherapy Process Scale (CPPS), where two independent raters scored 40 sessions (53). The STPP therapists scored significantly higher on the psychodynamic subscale than the CBT therapists (2.56 vs. 0.42), and the CBT therapists scored significantly higher on the cognitive-behavioral subscale than the STPP therapists (3.33 vs. 0.61) (32).

### 2.4 Psychometric instruments

#### 2.4.1 Childhood Trauma Questionnaire – Short Form

The CTQ-SF is a self-report inventory designed to assess childhood maltreatment retrospectively. It was developed by Bernstein et al. (54) to provide a brief yet reliable measure of adverse childhood experiences. It contains 28 items, including 25 items that assess trauma and three validity items (to detect response biases, such as minimization or denial). Each item is rated on a 5-point Likert scale ranging from 1 (never true) to 5 (very often true), yielding subscale scores from 5 to 25. Each CTQ-SF subscale score can be classified into four severity categories (none or minimal, low to moderate, moderate to severe, and severe to extreme) based on established cut-off values (54). The total CTQ-SF score, ranging from 25 to 125, is calculated as the sum of the five subscale scores.

Five types of childhood maltreatment by caregivers or significant others is evaluated by the CTQ-SF: emotional abuse, which involves verbal assaults, humiliation, or emotional manipulation; physical abuse, referring to physical harm or the threat of harm; sexual abuse, encompassing unwanted sexual contact or coercion; emotional neglect, characterized by a failure of caregivers to meet emotional needs or provide emotional support; and physical neglect, defined as a lack of basic physical necessities such as food, safety, or medical care.

The Norwegian version has previously exhibited good reliability and satisfactory accuracy to assess different types of childhood trauma (51). The CTQ-SF showed acceptable internal consistency in the current sample, measured by Cronbach’s alpha, for emotional abuse (α = 0.74), emotional neglect (α = 0.94), physical abuse (α = 0.72), and physical neglect (α = 0.73); good consistency for the total score (α = 0.89) and minimization-denial (α = 0.84); and excellent consistency for sexual abuse (α = 0.97).

#### 2.4.2 Hamilton Depression Rating Scale

The severity of depression was assessed by an observer using the 17-item Hamilton Depression Rating Scale (HDRS) at baseline and after 28 weeks (55). Each item measures symptoms experienced by the patient over the past week, including depressed mood, feelings of guilt, suicidal thoughts, insomnia, agitation, and weight loss. Items are rated on a scale of 0–4 or 0–2, depending on the symptom, with the total score reflecting the overall severity of depression. Scores are typically categorized as follows: 0–7 (normal), 8–13 (mild depression), 14–18 (moderate depression), 19–22 (severe depression), and ≥ 23 (very severe depression). To evaluate the reliability of the HDRS, we calculated the intraclass correlation coefficient (ICC 2.K) (56). Four raters, blinded to therapy allocation and treatment outcome, independently assessed the same 10 patients by viewing video recordings of the clinical interviews. The reliability coefficient was 0.96 for absolute decision, indicating excellent interrater reliability. The HDRS demonstrated acceptable internal consistency in the current sample, with α = 0.70 for the total score.

#### 2.4.3 The Beck Depression Inventory-II

Depression severity was also assessed through self-report using the Beck Depression Inventory-II (BDI-II) at baseline, 8 weeks, 16 weeks, and 28 weeks (57). The BDI-II consists of 21 items designed to evaluate a range of depressive symptoms experienced during the preceding two weeks, including sadness, pessimism, guilt, fatigue and suicidal ideation. Patients rate the severity of each symptom on a scale from 0 to 3, with higher scores indicating greater symptom severity. The total score is calculated by summing the responses across all items, with the following cut-off scores: 0–13 (minimal depression), 14–19 (mild depression), 20–28 (moderate depression), and 29–63 (severe depression). The BDI-II has also demonstrated excellent psychometric properties across diverse populations (58). When used alongside the HDRS, it provides complementary coverage of domains considered essential by patients experiencing depression, offering a comprehensive assessment of depressive symptoms (59). The internal consistency in the current sample was acceptable, with α = 0.77 for the total score.

### 2.5 Statistical analyses

All statistical analyses were performed using SPSS version 29.0. A modified intent-to-treat principle was applied, with all randomized participants analyzed if they had completed the CTQ-SF at baseline. Due to the low number of patients reporting physical abuse (n = 4) and sexual abuse (n = 9), these subtypes were omitted from the analysis.

All analyses were conducted with linear mixed models (LMM) using maximum likelihood estimation (60, 61). This approach does not require imputation, but instead includes all available observations for each participant and accounts for missing data under the missing at random (MAR) assumption. To evaluate whether the assumption of MAR was reasonable, we first compared baseline variables between patients with and without missing data using t-tests and chi-square tests. No significant differences were found in demographics (age, gender), treatment-related variables (previous treatment, antidepressant medication), baseline psychometric measures (HDRS, BDI-II, CTQ-SF), or proportions of moderate-severe abuse or neglect. We then conducted logistic regression analyses with missingness on BDI-II and HDRS at post-treatment as the dependent variables, and treatment group and CTQ-SF scores as predictors. Neither treatment group nor CTQ-SF predicted missingness. Taken together, these results support the MAR assumption.

For BDI-II, which was measured at four time points (baseline, 8 weeks, 16 weeks, and 28 weeks), time was coded as 0–2–4–7, while for HDRS, measured at two time points (baseline and 28 weeks), time was coded as 0–1. Model selection compared a simple linear regression (“baseline model”) to more complex models using the −2 Log Likelihood (LLH) and Akaike’s Information Criterion (AIC) as fit indices. Because AIC penalizes additional parameters, it was given the greatest weight when evaluating competing models relevant to the research hypotheses. To maintain readability, only statistically relevant values are reported in the results.

We developed six separate LMMs to address the three research questions (three models × two outcome measures). For readability, results from all models are presented together in a single table for each outcome. In these tables, the CTQ-SF main effect corresponds to research question 1 (baseline severity), the CTQ-SF × Time interaction to question 2 (prediction of outcome), and the CTQ-SF × Time × Intervention interaction to question 3 (moderation of treatment effects).

For the first research question, we tested whether higher CTQ-SF scores were associated with greater baseline depressive severity (BDI-II and HDRS separately). The models included CTQ-SF as a fixed effect and a random intercept to account for individual differences (covariance structure: Identity). We estimated this within the same mixed-effects framework used for the longitudinal analyses by coding time so that baseline = 0. This approach maintained a unified modeling strategy across aims and outcomes. For the second research question, we examined whether CTQ-SF predicted overall symptom change by including Time, CTQ-SF, and their interaction (CTQ-SF × Time) as fixed effects. For BDI-II, both a random intercept and a random slope for Time were specified to capture variability in individual symptom trajectories; for HDRS, only a random intercept was included because there were only two measurement points. An ARH (1) covariance structure was used for BDI-II to allow unequal variances and covariances over time, whereas a simplified AR (1) structure was applied for HDRS to improve convergence. For the third research question, we tested whether CTQ-SF moderated the relative effectiveness of CBT versus STPP using the three-way interaction term (CTQ-SF × Time × Intervention). Fixed effects were Time, Intervention (coded 0 = CBT, 1 = STPP), CTQ-SF (continuous), and all lower-order interactions. Random intercepts and slopes for Time were included for BDI-II; HDRS models only used a random intercept. Covariance structures matched those in the second research question. Additional covariates (treatment length, prior psychotherapy experience, antidepressant use, and baseline severity) were evaluated but did not improve model fit and were excluded. *Post hoc* graphical analyses were conducted to aid interpretation of any significant interactions.

To explore whether patients with severe childhood trauma differed from those without, we dichotomized the sample based on CTQ-SF scores. Those scoring in the severe range on at least one subscale were classified as having “severe CT” (N = 17), while the remaining patients were categorized as having “no severe CT” (N = 82). The two groups did not differ significantly on any demographic or clinical variables. In the comparison between patients with and without severe CT, we calculated odds ratios using cross-tabulation, and Fisher’s Exact Test was used to assess the statistical significance of differences in response and remission rates between the groups. Fisher’s exact test was chosen over logistic regression due to the small cell counts in several subgroups, which could lead to unstable estimates in regression models. Response was defined as a reduction of ≥50% in symptom scores from baseline to 28 weeks, and remission as HDRS score ≤7 or BDI-II score ≤9. Independent t-tests were used to compare patient characteristics between the two groups.

As multiple hypotheses were tested in this study, we considered the risk of false positives due to multiple comparisons. For the first and second hypotheses, no correction for multiple testing was applied. As discussed in the introduction, these hypotheses were grounded in prior research and therefore considered confirmatory rather than exploratory. In such cases, the risk of false positives is lower, while applying strict corrections could increase the risk of false negatives. For the third hypothesis, we applied false discovery rate (FDR) correction using the Benjamini-Hochberg procedure (62), given that these analyses were more exploratory (63).

## 3 Results

### 3.1 CTQ-SF and baseline severity

At baseline, patients with higher total CTQ-SF scores reported more depressive symptoms on BDI-II (E = 0.23, SE = 0.08, p = .003) (see **Table 2**). Among the subtypes of CT, emotional neglect (E = 0.36, SE = 0.15, p = .021) and physical neglect (E = 1.07, SE = 0.32, p = .001) were significantly associated with higher baseline BDI-II scores. Emotional abuse was not significantly associated with baseline depression severity. Patients with higher total CTQ-SF also reported more depressive symptoms on HDRS (E = 0.11, SE = 0.05, p = .018) (see **Table 3**), but none of the subtypes were significantly associated with higher baseline HDRS scores.

**Table 2:** Results from the linear mixed-effects models examining the change in depressive symptoms over time in short-term psychodynamic psychotherapy (STPP) and cognitive behavioral therapy (CBT), measured by the Beck Depression Inventory-II (BDI-II).

	Estimate	SE	95% CI	T-value	P-value
CTQ-SF Total					
CTQ-SF	0.23	0.08	0.08 – 0.38	3.09	0.003
CTQ-SF x Time	0.02	0.02	-0.02 – 0.05	0.93	0.357
CTQ-SF x Time x Intervention	0.04	0.03	-0.02 – 0.10	1.41	0.161
CTQ-SF Emotional abuse					
CTQ-SF	0.22	0.24	-0.26 – 0.70	0.90	0.369
CTQ-SF x Time	0.05	0.05	-0.06 – 0.15	0.89	0.375
CTQ-SF x Time x Intervention	0.16	0.09	-0.03 – 0.34	1.71	0.090
CTQ-SF Emotional neglect					
CTQ-SF	0.36	0.15	0.05 – 0.66	2.34	0.021
CTQ-SF x Time	0.00	0.03	-0.06 – 0.07	0.06	0.950
CTQ-SF x Time x Intervention	0.03	0.06	-0.09 – 0.16	0.51	0.611
CTQ-SF Physical neglect					
CTQ-SF	1.07	0.32	0.44 – 1.70	3.34	0.001
CTQ-SF x Time	0.07	0.08	-0.10 – 0.23	0.79	0.431
CTQ-SF x Time x Intervention	0.22	0.16	-0.09 – 0.53	1.42	0.160

CTQ-SF, Childhood Trauma Questionnaire – Short Form. P-values from Type III tests of fixed effects. Intervention coded: 0 = CBT, 1 = STPP. Time coded 0–2–4–7.

**Table 3:** Results from the linear mixed-effects models examining the change in depressive symptoms over time in short-term psychodynamic psychotherapy (STPP) and cognitive behavioral therapy (CBT), measured by the Hamilton Depression Rating Scale (HDRS).

	Estimate	SE	95% CI	T-value	P-value
CTQ-SF Total					
CTQ-SF	0.11	0.05	0.02 – 0.21	2.39	0.018
CTQ-SF x Time	0.04	0.07	-0.10 – 0.17	0.52	0.606
CTQ-SF x Time x Intervention	0.17	0.11	-0.05 – 0.39	1.53	0.130
CTQ-SF Emotional abuse					
CTQ-SF	0.26	0.15	-0.04 – 0.55	1.73	0.085
CTQ-SF x Time	0.07	0.21	-0.35 – 0.49	0.33	0.742
CTQ-SF x Time x Intervention	0.69	0.33	0.04 – 1.35	2.11	0.038
CTQ-SF Emotional neglect					
CTQ-SF	0.18	0.10	-0.01 – 0.37	1.90	0.060
CTQ-SF x Time	0.00	0.14	-0.27 – 0.28	0.038	0.970
CTQ-SF x Time x Intervention	0.08	0.23	-0.37 – 0.53	0.36	0.724
CTQ-SF Physical neglect					
CTQ-SF	0.29	0.21	-0.12 – 0.70	1.38	0.168
CTQ-SF x Time	0.04	0.33	-0.61 – 0.70	0.13	0.894
CTQ-SF x Time x Intervention	0.56	0.58	-0.60 – 1.72	0.96	0.341

CTQ-SF, Childhood Trauma Questionnaire – Short Form. P-values from Type III tests of fixed effects. Intervention coded: 0 = CBT, 1 = STPP. Time coded 0–1.

### 3.2 CTQ-SF as predictor of treatment outcome

For BDI-II, the two-way interaction between CTQ-SF scores and time (CTQ-SF x Time) was not significant for total CTQ-SF score (E = 0.02, SE = 0.02, p = .357) or any subtypes (see **Table 2**). This indicates that the rate of symptom improvement over time was not significantly influenced by the severity of childhood trauma. The same was found for HDRS, where neither total CTQ-SF score (E = 0.04, SE = 0.07, p = .606) nor any subtypes were significant predictors of symptom trajectories (see **Table 3**).

Patients with severe CT (N = 17) had a mean age of 32.2 years, compared to 31.0 years in the non-severe group. The proportion of females was 64% in the severe CT group and 58% in the non-severe group. Antidepressant use was roughly similar (41% vs. 43%), as was the prevalence of personality disorders (35% vs. 26%). Previous psychotherapy experience was more prevalent in the severe CT group (76% vs. 60%), though the difference was not statistically significant. While HDRS scores showed no significant difference between groups (mean 19.8 vs. 17.7; p = .130), BDI-II scores were higher in the severe CT group (mean 30.7 vs. 26.9), with a non-significant difference (p = .057).

To explore whether severe CT was associated with poorer treatment outcomes, we compared response and remission rates between patients with and without severe CT. For BDI-II, response rates were 33% vs. 58% (OR = 0.38, CI: 0.11–1.39, p = .211), and remission rates were 17% vs. 37% (OR = 0.32, CI: 0.06–1.56, p = .201). HDRS outcomes showed a similar pattern, with lower response (36% vs. 42%, OR = 0.79, CI: 0.21–2.95, p = .390) and remission rates (18% vs. 33%, OR = 0.44, CI: 0.09–2.23, p = .498) in the severe CT group. However, there were no significant differences between the groups in either response or remission rates.

### 3.3 CTQ-SF as moderator of treatment effectiveness

For total CTQ-SF scores, the three-way interaction (CTQ-SF x Time x Treatment) was non-significant for both BDI-II (E = 0.04, SE = 0.03, p = .161) (see **Table 2**) and HDRS (E = 0.17, SE = 0.11, p = .130) (see **Table 3**). This indicates that the overall severity of childhood trauma did not moderate the relative effectiveness of CBT and STPP in reducing depressive symptoms. No significant moderation effects were found for any subtypes on either BDI-II or HDRS. The only exception was emotional abuse on HDRS, which initially showed a significant moderating effect (E = 0.69, SE = 0.33, p = .038). Upon visual inspection, this effect appeared to be mostly driven by patients with severe levels of emotional abuse. Including the three-way interaction between CTQ-SF, time, and treatment improved the overall fit of the model, as the final model had a lower AIC (1109.1 vs. 1111.3) and a higher log-likelihood (–1089.1 vs. –1093.3). A likelihood ratio test confirmed that this improvement was statistically significant. This supports that emotional abuse may be associated with differential treatment response over time. However, when applying the Benjamini-Hochberg procedure to control the false discovery rate at the 0.05 level, the critical p-value for significance was 0.0071. As a result, the effect of emotional abuse did not remain statistically significant after adjusting for multiple testing.

## 4 Discussion

This study explored how childhood trauma affects the outcome of CBT and STPP in treating MDD. We hypothesized that higher CTQ-SF scores would be associated with greater baseline depressive severity, predict poorer treatment outcomes, and moderate the relative effectiveness of CBT and STPP, favoring the latter. Our findings provided mixed support for these hypotheses.

In line with our expectations, higher CTQ-SF scores were associated with increased baseline depressive severity. This aligns with previous research highlighting the impact of childhood trauma on the severity of depressive symptoms (3). Among the subtypes, we found fewer significant associations in the observer-rated HDRS than the self-reported BDI-II. For example, emotional abuse was significantly associated with more depressive symptoms only on BDI-II. One could speculate that the effects of emotional abuse, a trauma subtype often linked to negative self-concepts (64), could more easily be picked up by the self-reported BDI-II than clinician-rated HDRS. Their differences in symptom focus may also partly explain the discrepancy (59), as the HDRS emphasizes severe neurovegetative and psychomotor symptoms, while the BDI-II captures more cognitive and affective dimensions of depression, such as self-criticism and guilt, which may be more pronounced in patients with a history of emotional abuse (17). The strong association between self-reported depressive symptoms and CTQ scores may partly reflect shared method variance, i.e., the tendency that different constructs measured with the same medium (self-report) may produce artifactual covariance independent of the content of the constructs themselves (65). To better understand these discrepancies, future research should continue to incorporate both types of measures.

Contrary to our hypothesis that CTQ-SF scores would predict poorer treatment outcomes, we found no significant associations between CTQ-SF severity and the degree of symptom improvement in either CBT or STPP. Overall, these findings suggest that the severity of childhood trauma does not substantially alter the effectiveness of CBT or STPP, aligning with recent research (18). This indicates that standard short-term treatments for depression remain viable options for patients with depression and CT. Previous research, however, has also shown that patients with CT experience more residual symptoms at post-treatment, and are less likely to achieve full remission, increasing their risk of relapse (5). Our results show a similar trend, with numerically lower response and remission rates in the severe CT group, although these differences were not statistically significant. It remains unclear whether this small difference might translate into a higher relapse rate for patients with severe CT compared to those without, underscoring the need for more research.

We hypothesized that CTQ-SF scores would moderate the relative effectiveness of CBT and STPP, but our findings did not reveal significant differences. The only moderation effect was for emotional abuse, which was associated with better outcomes in STPP compared to CBT on HDRS. This effect was limited to the HDRS and did not remain significant after correction. Therefore, it should be interpreted with caution, as it was the only positive finding among all trauma subtypes examined. The lack of robust moderating effects contrasts with some previous studies. Krakau et al. (39) found that psychoanalytic therapy was more effective than CBT in long-term treatment, attributing this to the ability of long-term psychodynamic therapy to activate and process unconscious childhood memories. If this interpretation holds, it may simply be that the short duration of STPP in our study was insufficient to activate these processes. However, this explanation does not fully align with findings from Heinonen et al. (41), who reported that childhood trauma did not predict worse outcomes in STPP compared to long-term therapy over five years. Our results also contrast Harkness et al. (40), who reported that CBT was more effective than IPT for depressed patients with a history of childhood trauma, though their effect was limited to individuals with severe maltreatment. IPT focuses more on current interpersonal roles and communication patterns than childhood experiences (66), and this limited focus on past relational trauma may make IPT less effective for patients with CT than STPP.

Several limitations of the present study warrant careful consideration. The trial did not include a pharmacotherapy-only control group or a waitlist control, so we cannot evaluate the effects of psychotherapy relative to no active treatment or to medication alone. This was a deliberate design choice, as the primary aim was to compare two active psychotherapy approaches within routine clinical practice. Another limitation is that patients with bipolar I disorder were excluded. This decision reduced diagnostic heterogeneity and allowed a clearer focus on unipolar depression, but it also limits generalizability across the broader mood disorder spectrum. Childhood trauma was measured retrospectively, which may be influenced by recall bias. Retrospective reports can also be shaped by current mood or other psychological factors, preventing strong conclusions about causality. This may have influenced baseline severity associations in particular, and should be considered when interpreting our findings. Only three patients in our sample met the criteria for post-traumatic stress disorder (PTSD). Thus, our findings primarily apply to patients with depression and CT without comorbid PTSD, and caution is warranted in generalizing to patients with PTSD. Due to the small number of patients reporting sexual and physical abuse, these subtypes were not analyzed separately to avoid unstable estimates. The present analysis, focusing specifically on CT, should be regarded as exploratory, since it was not separately pre-registered with specified hypotheses and methods. This is consistent with the overall exploratory design of the RCT. As noted in the trial protocol (48), statistical power for detecting moderation effects was expected to be limited, which is common in psychotherapy RCTs involving multiple potential moderators. No separate power analysis was conducted for childhood trauma as a moderator. However, the sample size is sufficient to detect moderate-sized effect sizes. Thus, the findings suggest that if differences in treatment effects exist for patients with a history of childhood trauma, they are unlikely to be large. However, the small size of the subgroup with severe CT substantially limits statistical power and increases the risk of false negatives. While no significant effects were found, the results should be interpreted cautiously, as small-to-moderate effects may have gone undetected. Another limitation is that pharmacological regimens were managed by treating clinicians and allowed to vary during the trial, consistent with routine practice. As both treatment arms were equally exposed to such changes, systematic bias in the comparison between CBT and STPP is unlikely. It is also important to note that the present study compared standard CBT and STPP, which are not specifically designed as trauma-focused interventions. Adaptations of these therapies that directly target traumatic experiences may yield different outcomes in patients with CT.

Future research should examine whether CT moderates psychotherapy outcomes in larger samples with sufficient statistical power to detect small-to-moderate effects. Longer follow-up periods are also needed to determine whether trauma influences relapse risk and the development of treatment-resistant depression (TRD) (67). CT has been associated with more chronic illness courses and may contribute to TRD (68). A recent editorial emphasizes the substantial burden and therapeutic challenges of TRD (69), reinforcing the importance of psychotherapeutic interventions for patients with CT that prevent chronicity. Expert consensus further highlights that the clinical management of TRD requires a wide variety of approaches (68), and additional research is needed to clarify the most effective strategies. While our study focused on psychotherapeutic interventions, it is important to situate these findings within a broader treatment landscape. Recent work has pointed to novel pharmacological augmentation strategies in treatment-resistant conditions (70). In line with the trend toward personalization, recent case studies suggest that ketamine treatment might be especially beneficial for patients with a depersonalized depression subtype (71), which could be more common in patients with trauma (72).

## 5 Conclusions

This study demonstrates that both CBT and STPP are effective treatments for depressed patients with a history of childhood trauma, with no substantial differences in their short-term effectiveness. Although patients with trauma presented with greater baseline severity, they improved to a similar degree as those without trauma. These findings suggest that childhood trauma should not be regarded as a barrier to improvement in short-term psychotherapy. Both CBT and STPP appear to be viable treatment options, and the choice between them may be guided more by patient preference, therapist expertise, and service availability than by trauma status alone.

## Data Availability

The raw data supporting the conclusions of this article will be made available by the authors, without undue reservation.
